# Biomaterials for corneal endothelial cell culture and tissue engineering

**DOI:** 10.1177/2041731421990536

**Published:** 2021-02-16

**Authors:** Mohit Parekh, Vito Romano, Kareem Hassanin, Valeria Testa, Rintra Wongvisavavit, Stefano Ferrari, Atikah Haneef, Colin Willoughby, Diego Ponzin, Vishal Jhanji, Namrata Sharma, Julie Daniels, Stephen B Kaye, Sajjad Ahmad, Hannah J Levis

**Affiliations:** 1 Faculty of Brain Sciences, Institute of Ophthalmology, University College London, London, UK; 2 International Center for Ocular Physiopathology, Fondazione Banca degli Occhi del Veneto Onlus, Venice, Italy; 3 St. Paul’s Eye Unit, Royal Liverpool Broadgreen University Hospital, Liverpool, UK; 4 Instituto Universitario Fernandez-Vega, Universidad de Oviedo and Fundacion de Investigacion on Oftalmologica, Oviedo, Spain; 5 Department of Eye and Vision Science, Institute of Ageing and Chronic Disease, University of Liverpool, Liverpool, UK; 6 Eye Clinic, Department of Neuroscience, Rehabilitation, Ophthalmology, Genetics, Maternal and Child Health, University of Genoa, Genoa, Italy; 7 Ospedale Policlinico San Martino IRCCS, Genoa, Italy; 8 HRH Princess Chulabhorn College of Medical Sciences, Chulabhorn Royal Academy, Bangkok, Thailand; 9 School of biomedical sciences, University of Ulster, Belfast, UK; 10 Department of Ophthalmology, University of Pittsburgh, Pittsburgh, PA, USA; 11 Dr. Rajendra Prasad Centre for Ophthalmic Sciences, All India Institute of Medical Sciences, New Delhi, India; 12 Moorfields Eye Hospital NHS Trust Foundation, London, UK

**Keywords:** Cornea, tissue engineering, biomaterials, cell culture, endothelial cells

## Abstract

The corneal endothelium is the posterior monolayer of cells that are responsible for maintaining overall transparency of the avascular corneal tissue via pump function. These cells are non-regenerative in vivo and therefore, approximately 40% of corneal transplants undertaken worldwide are a result of damage or dysfunction of endothelial cells. The number of available corneal donor tissues is limited worldwide, hence, cultivation of human corneal endothelial cells (hCECs) in vitro has been attempted in order to produce tissue engineered corneal endothelial grafts. Researchers have attempted to recreate the current gold standard treatment of replacing the endothelial layer with accompanying Descemet’s membrane or a small portion of stroma as support with tissue engineering strategies using various substrates of both biologically derived and synthetic origin. Here we review the potential biomaterials that are currently in development to support the transplantation of a cultured monolayer of hCECs.

## Introduction

### Corneal endothelial dysfunction

**Figure 1.**
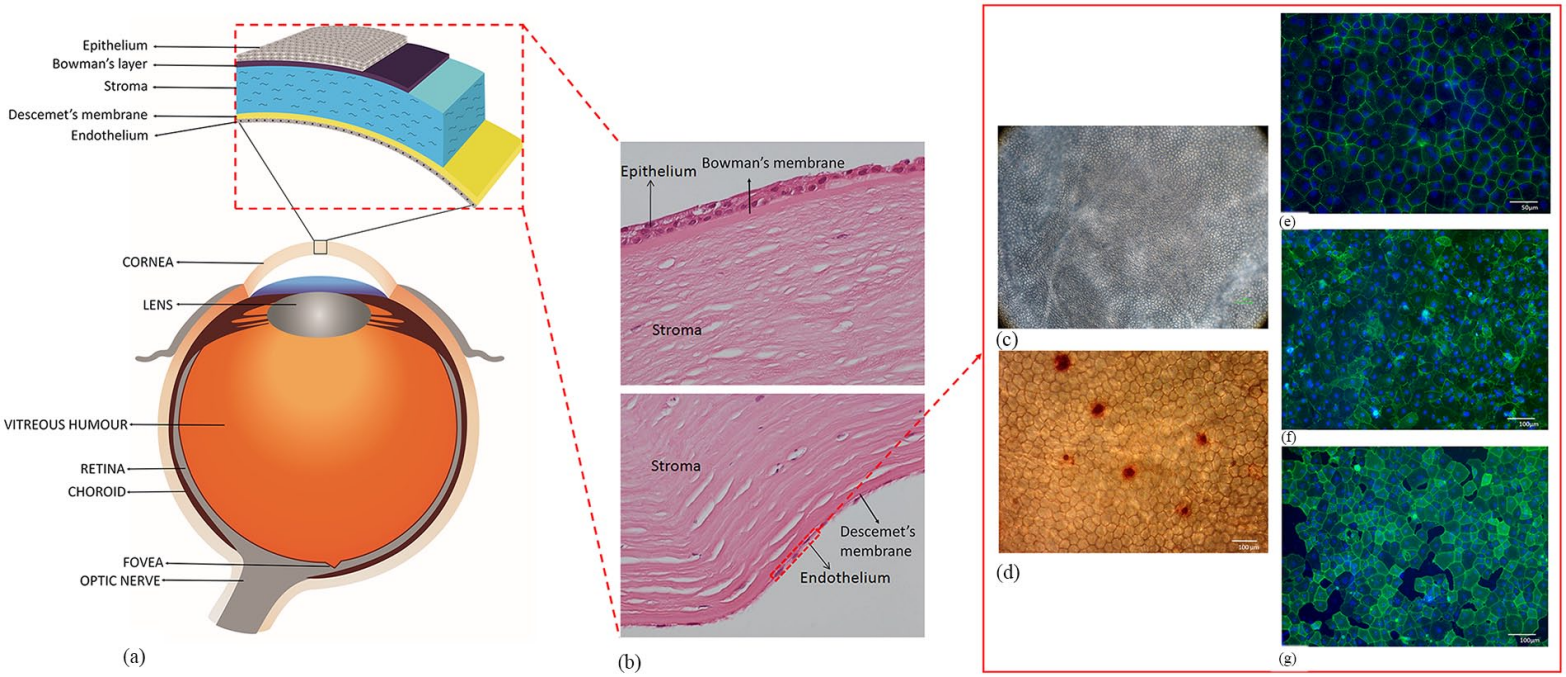
Anatomy of the human eye and cornea: (a) The figure illustrates different parts of the eye and layers of the corneal tissue, (b) histological section of human cornea showing different layers of the tissue, (c) corneal endothelial cell viability using trypan blue staining observed under light microscope and (d) alizarin red staining showing the hexagonality of endothelial cells. Biomarkers such as (e) ZO-1, (f) Tag-1A3, and (g) Tag-2A12 to determine the presence of specific proteins like tight-junction and surface epitopes. These biomarkers are used on cultured corneal endothelial cells to characterize the cell type and for functional analysis.

The cornea is a transparent avascular tissue forming the anterior part of the eye.^1^ It plays a critical role in transmitting and focusing incoming light onto the retina (Figure 1(a)).^2^ It consists of five layers with the corneal endothelium (CE) being the posterior monolayer (Figure 1(b)).^3^ Viability of the CE is crucial for maintaining corneal transparency^4^ which is preserved through dynamic regulation of corneal hydration between a “leaky” barrier and active ionic pumps on the corneal endothelial cells (CECs).^5^ The viability using trypan blue staining determine the suitability of the graft for transplantation (Figure 1(c)). However, hexagonality using alizarin red stain (Figure 1(d)) along with certain biomarkers such as ZO-1 (Figure 1(e)), Tag-1A3 (Figure 1(f)) and Tag-2A12 (Figure 1(g)) are some of the parameters that are used for characterization of CECs. Damage to these cells may result in loss of transportation of ions and solutes leading to corneal oedema.^6,7^ As the CECs do not possess regenerative capability in vivo, their health needs to be retained throughout life to prevent the development of corneal oedema.^8^ Endothelial dysfunction is the most common indication for corneal transplantation.^9^ The main causes of endothelial dysfunction are the corneal dystrophies, which are usually bilateral, symmetric, slowly progressive, and not related to environmental or systemic factors.^10^ The most commonly observed dystrophy is Fuchs’ endothelial corneal dystrophy (FECD) accounting for approximately 39% of the global corneal transplants undertaken.^9^ Degeneration of the CE is heritable and mutations in seven genes (AGBL1, COL8A2, LOXHD1, SLC4A11, TCF4, ZEB1, DMPK) have shown to be causal or highly associated with FECD.^11,12^ In terms of acquired causes, pseudophakic bullous keratopathy is considered to be the predominant cause of endothelial failure.

### Current treatment strategy

**Figure 2.**
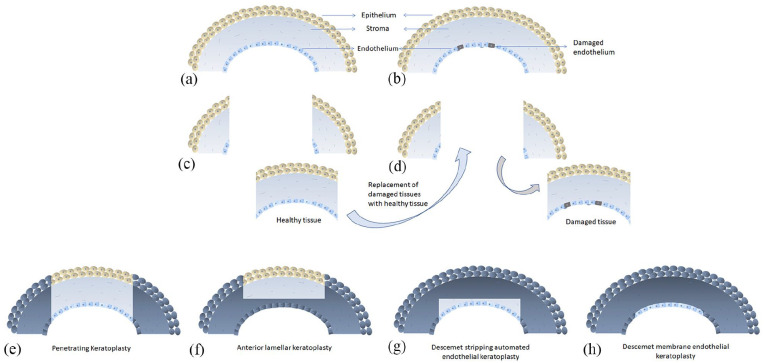
Illustration of conventional corneal transplantation technique: (a) normal cornea, (b) cornea with diseased or dysfunctional endothelium, (c) part of a healthy donor tissue is replaced with (d) part of damaged recipient tissue. Representation of conventional full thickness (e) penetrating keratoplasty and selective modern transplantation procedures such as (f) anterior lamellar keratoplasty, (g) Descemet’s stripping automated endothelial keratoplasty, and (h) Descemet’s membrane endothelial keratoplasty.

The gold standard treatment for corneal endothelial disease or dysfunction is corneal transplant (Figure 2) where a diseased corneal endothelium is replaced with a healthy donor corneal endothelium (Figure 2(a)–(d)). When the technique was first introduced, full thickness corneal replacement was a standard practice, known as a penetrating keratoplasty (PK) (Figure 2(e)), and is still used along with anterior lamellar keratoplasty (Figure 2(f)) in cases where stromal scarring is present. In cases where the stroma is unaffected and only the endothelial layer is defective than a lamellar technique can be used. These include the Descemet’s stripping automated endothelial keratoplasty (DSAEK) (Figure 2(g)) and more recently the Descemet’s membrane endothelial keratoplasty (DMEK; Figure 2(h)). Both techniques involve the transplantation into the anterior chamber of a healthy monolayer of hCECs from a cadaveric donor along with either just the Descemet’s membrane (DM) or the DM and a portion of stroma (usually <100 µm). This layer is then attached to the posterior surface of the cornea (recipient DM and endothelial layer removed) with an air tamponade. The DMEK procedure is favored by some surgeons because of the faster recovery of visual acuity but the drawback is that rebubbling rates, due to graft detachment, can be higher in DMEK than in DSAEK.^13–15^

### New approaches to corneal endothelial replacement

A key issue with corneal transplantation is the limited availability of suitable donor tissue; the number of patients that require treatment is much greater than the number of donors available.^9^ This is especially true in developing countries where cadaveric donation is limited due to absence of eye bank facilities, religious and cultural factors, and lack of knowledge about donation.^16,17^ Even when donation is high in a particular region, approximately one third of harvested donor tissues are not suitable for transplant due to low endothelial cell count or presence of infectious agents upon screening.^9^ Another limitation of the cadaveric donor transplantation is the long-term risk of allogenic graft rejection and failure.^18^ Transplant failure is often due to loss of hCECs from the donor layer. It has been reported that 30% of hCECs are lost within the first 6 months of transplantation.^19^ There is an opportunity to develop biomaterials that not only serve as a scaffold for hCEC transplantation but also may enhance cell function to increase the long-term success of transplanted grafts or even improve the in vitro expansion success of hCECs. Biomaterials for tissue engineering can be characterized in a number of ways. Here we classify them based on their origin into biologically derived, synthetic, and semi-synthetic.

## Isolation and expansion of hCECs

Cell- based therapies for the cornea are characterized by the isolation of single cells from a cadaveric donor cornea, usually using enzymatic digestion, to allow expansion of their number in vitro. A commonly used method optimized by Peh et al.^20^ involves removing the DM and placing this into collagenase to lift the cells from the DM, followed by Tryple Express to break up the cell clumps to leave a single cell suspension. The single cells are then collected and plated onto fibronectin collagen and albumin (FNC) coating mix coated plates in medium that enhances attachment. This medium is then changed after cells have attached to one that promotes expansion before being placed back into the original medium to maintain their phenotype after about 2 weeks in culture. Cells expand in number over time and then can be harvested for use to treat multiple patients rather than the usual 1:1 ratio that is seen for most cadaveric corneal donations.

### Injected cell therapy

One recent development in the attempt to produce a new therapy for CEC replacement is the CEC-injection approach pioneered by Kinoshita and his colleagues.^21–24^ In this approach the cultivated CECs are directly injected into the anterior chamber of the recipient. The patient must then remain face down for 2–3 h to allow the cells to attach to the recipient posterior cornea. A very recent study was published reporting a 5 year follow up of 11 patients who were treated with a CEC injection in a first-in-human study.^25^ The results were promising, showing that the best corrected visual acuity (BCVA) was significantly improved in 10 treated eyes and no adverse reactions to the hCEC injection were observed. Importantly, the authors claim that from one donor cornea, at least 300 eyes can be treated using this therapy. It is worth noting, however, that the DM of the patients was not intentionally removed, only abnormal materials on the host DM were mechanically removed in an 8 mm diameter central area. This is because it has been found that the injected CECs do not form a functional monolayer and are unable to improve corneal transparency or reduce corneal thickness in a rabbit model of corneal damage when directly injected onto the bare stroma after DM removal.^26^ It is likely that they undergo endothelial mesenchymal transition (EMT) and do not form a complete, functional barrier. The FECD cases in the study (n=7) all show the remaining presence of corneal guttae even after 5 years. One limitation of this therapy, therefore, is that it would not be suitable for patients that have extensive guttae in the central field of vision as it has been shown that corneal guttae, even without oedema, cause the quality of vision to deteriorate due to intraocular forward scatter resulting in visual disturbances.^27^

Harvesting and expanding CECs to gain sufficient numbers for cell or tissue engineering therapies is not easy and can be very donor dependent; younger donors can be more easily expanded in vitro although methods have been devised to encourage the expansion of older donors using techniques such as forced adherence.^28^ Consequently, numerous investigations have been undertaken to optimize the expansion procedure, many using different biomaterials as culture substrates, summarized in Table 1.

**Table 1.** Summary of the advantages and limitations of each material for the two different applications of hCEC culture and scaffold based tissue engineering for the corneal endothelium.

hCEC culture	Category	Type	Advantages	Limitations	In vivo model using hCECs
	Biologically derived	Extracellular matrix proteins^29,30^ (collagens (I, IV),^31^ fibronectin, laminin,^32,33^ FNC coating, whole ECM)^34^	Mimic components of DM, improve adhesion and proliferation, maintain phenotype	No consensus on best one, can be animal derived and not well defined	N/A
	Synthetic	Temperature responsive polymers (poly(N-isopropylacrylamide) (PIPAAm))^35–39^	Stable, well defined, whole cell sheet recovered	Fragile, requires another biomaterial for transplant, effect of temp. change on cells not known	Rabbit
	Semi-synthetic	Chemo-mechanical biomimetics (Polydimethylsiloxane based)^40^	Proliferation and phenotype of cells maintained, mimics basement membrane	Not easy to scale up	N/A
Scaffold based tissue engineering	Category	Type	Advantages	Limitations	In vivo model using hCECs
	Biologically derived	Amniotic membrane^41–46^	Inert, non-cytotoxic, low rejection, proven biocompatibility in the eye	Availability from donor bank, reproducibility, lack of mass manufacturing, sub-optimal transparency, variable rate of biodegradability, risk of contamination and transmission of infectious diseases	Rabbits, cats
		Silk Fibroin^47–53^	Low immunogenicity, good transparency, non-toxic, controlled degradation rate	Low elasticity and mechanical strength, fragile, difficult to handle and manufacture, decreased biocompatibility, and increased hypersensitivity	Rabbit
		Human anterior lens capsule^54,55^	Capability to maintain intact barrier, good adhesion to bare stroma, bio-mechanical properties similar to DMEK grafts	Sourced from cadaveric donors, reproducibility, lacks mass manufacturing potential	Pigs
		Decellularized corneas^56–58^	Capability to maintain intact barrier and ionic pump function, retains transparency, ultrastructure, mechanical properties	Can be a lengthy process, dependent of the availability of the tissues, no data on human corneas	Rabbit
		Fish scales^59–62^	Cytocompatible, transparency, good availability	Cell proliferation poor, low adherence, irregular topography	In vitro only
Scaffold based tissue engineering	Category	Type	Advantages	Limitations	In vivo model using hCECs
		Plastic compressed collagen^63–64^	Rapid scalable production, improved mechanical strength, capability to maintain intact barrier	Transparency not optimal, no in vivo studies	Porcine *ex vivo*
		Gelatin^65–67^	Low antigenicity, maintains cell binding motifs, inexpensive, widely availability, permeability to water, transparency, elastic modulus, porosity, good cell adherence	Less relative modifiability compared to synthetic polymers, lack of stability as corneal graft, unknown cytocompatibility with hCECs	Monkey, rabbit
		Chitosan blends^68^	Biocompatibility and biodegradability	Low strength, induces inflammation	Rabbit
	Synthetic	Poly-ε-lysine peptide hydrogel^69^	Good cell adhesion and growth, properties can be controlled for customized manufacturing, good mechanical properties and transparency, can be functionalized with synthetic peptides	No in vivo studies or use with hCECs	Porcine *ex vivo* - HCEC-12 and porcine cells
		Poly(methyl-methacrylate)^70,71^	Moderately inexpensive, modifiability of transparency	Cytotoxic, low viscosity	In vitro only
		Poly(lactic-co-glycolic acid)^70^	Biocompatible, FDA approved for many applications	Expensive, no in vivo studies	In vitro only
		Polycaprolactone^71^	Biocompatible, maintenance of hCEC morphology, fibers can be aligned	No in vivo studies	In vitro only
	Semi-synthetic	Gelatin methacrylate (GelMA+)^19,72^	Increased mechanical strength, maintains cell binding motifs, cytocompatible, increased modifiability compared to gelatin	Expensive and lengthy production process, no in vivo studies	In vitro only
		Chitosan and polycaprolactone^73,74^	Biodegradable, FDA approved, mechanical property, promote cell adhesion and ECM production, functional maintenance of cells	No in vivo studies	In vitro only
		Chitosan and PEG^75^	Tensile strain and stress similar or greater to cornea, good transparency, good cell attachment and proliferation	No in vivo studies	Sheep
Emerging technologies		Electrospun/electrosprayed^76,77^	Transparency, cytocompatibility, bioactive compounds can be incorporated	Limited studies	In vitro only
		3D bioprinting^78–81^	CECs survive the process, graft size can be chosen	Other materials still required as base, hCEC cells not confluent after 10 days	Rabbit

### Biologically derived materials

**Figure 3.**
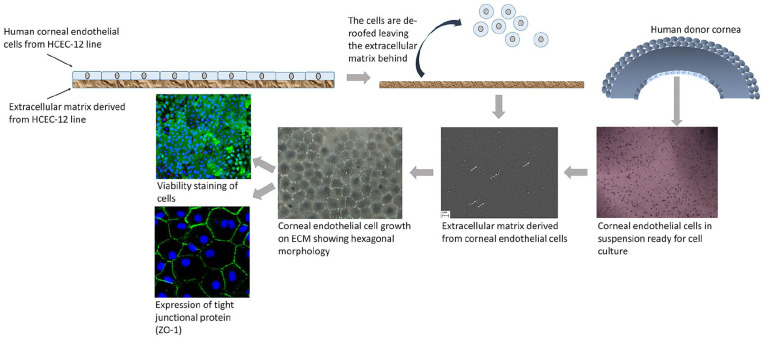
Human corneal endothelial cell culture on HCEC-12 derived extracellular matrix (ECM). HCEC-12 cells are cultured on a culture plate. ECM is laid by the HCEC-12 cells naturally. Upon confluence, the cells are detached leaving behind the ECM. Corneal endothelial cells from human donor corneas are isolated and cultured on the ECM. White arrows show fiber-like collagen structures and dotted white arrow show cell debris. Cell morphology, viability and expression of tight-junction protein are checked to confirm the health and for end-stage characterization.

Extracellular matrix proteins are particularly important in terms of attachment and expansion of hCECs in culture as well as a potential coating on materials for transplantation. The most common and extensively studied ECM proteins are collagens, fibronectin and laminins. In order to assess the effects of these proteins on the hCECs, tissue culture plates are coated with ECM proteins, either one or a combination of proteins (i.e. FNC Coating Mix^®^—fibronectin, collagen, and albumin) and then compared to uncoated plates. One study that looked at a number of factors that may affect the success of isolation and culture of CECs included a study on ECM proteins.^29^ They concluded that FNC coating was the best surface with 56% of cultures grown on these plates being scored a success compared to only 23% from fibronectin, and 8% grown on collagen IV. The results of another study suggested that fibronectin overall performed the best. All proteins (collagen I, collagen IV, fibronectin, and FNC coating) except laminin increased hCEC adhesion compared to uncoated control.^30^ In fact, laminin-5 has shown mixed results; another study showed that there was a statistically significant increase in cellular proliferation and cellular adhesion on the laminin-5 coated surface compared to fibronectin, with similar adhesion to collagen IV.^31^ The conflicting results seen are perhaps due to different techniques and growth factors used in these studies but could also be related to the isoform of laminin used. A more recent study compared the effect on hCECs of coating with laminin-511, -521, and -211.^32^ They found that the -511 and -521 isoforms did increase the number of adherent and proliferating hCECs compared to control uncoated plates, whereas -211 has no effect. The 511 and 521 isoforms also maintained the cell density and functional phenotype of the CECs after over 100 days in culture. These two isoforms were also found to be expressed in the DM. Laminin-511 has also been shown to promote rapid adhesion, tight junction formation and expression of Na^+^/K^+^-ATPase in CECs injected directly into the anterior chamber of a rabbit model of endothelial disease.^33^ Laminin-511 was injected into the anterior chamber before injections of CECs and was found to settle to form a coating on the posterior surface of the DM. The corneal clarity in the laminin group was restored 7 days before the control group. Another study reported that when hCECs were cultured on ECM derived from human corneal endothelial cell line HCEC-12 (Figure 3), cell doubling time was significantly less in the whole ECM group compared to FNC control. This is because the secreted ECM retains the physiological environment, that is, proteins and growth factors that are required for the growth of hCECs.^34^ Although there are conflicting results related to the best ECM protein to coat for cell culture and expansion it is clear that an ECM protein is better than no coating. The choice will ultimately need to be optimized by the end user as it may be dependent on the donor material, and if used for coating a biomaterial for transplant, the characteristics of that particular material.

### Synthetic materials

The key to a useful biomaterial for CEC expansion is to provide a stable, easily usable polymer, which may have initial cell adhesion properties as well as allowing detachment of a cohesive monolayer when required. With respect to this, the most successful synthetic polymer to date is the temperature-responsive polymer poly(N-isopropylacrylamide) (PIPAAm), which is already being used clinically for corneal epithelial cell expansion.^35^ The regulation of the temperature in a controlled manner allows both the initial adhesion (37°C) and the latter detachment (20°C) of the cells from the PIPAAm.^36–39^ As highlighted in a review, a number of studies have shown that PIPAAm supports important structures such as the Na^+^/K^+^-ATPase pump and morphology of CECs with the presence of microvilli and cellular interconnections.^2^ Rabbit models have been used to assess cell functionality after culture on PIPAAm.^37–39^ The cell layers were retrieved from the PIPAAm surfaces and transferred to gelatin disc carriers for transplantation into the rabbit anterior chamber. Both, the clarity of the cornea and the corneal thickness returned to normal within 2 weeks and histological examination suggested the hCECs were spread over the DM with tight junction formation between cells. The effect of the temperature change on the bioactivity of hCECs remains to be investigated as does the implications of the degradation of the gelatin disc within the anterior chamber.

### Semi-synthetic materials

A study by Palchesko et al.^40^ showed that a bioengineered substrate recapitulating the chemo-mechanical properties of DM improved the in vitro expansion of CECs while maintaining phenotype. It was observed that the bovine CECs cultured on a polydimethylsiloxane surface with elastic modulus of 50 kPa and collagen IV coating achieved 3000-fold expansion. Cells grew in higher-density monolayers with polygonal morphology and ZO-1 localization at cell-cell junctions in contrast to control cells on tissue culture polystyrene that lost these phenotypic markers and showed increased α-smooth muscle actin expression and fibronectin fibril assembly. The results demonstrate that a biomimetic substrate presenting native basement membrane ECM proteins and mechanical cues may be a key element in optimizing the expansion of CECs for potential therapeutic applications.

## Scaffold based tissue engineered grafts

**Figure 4.**
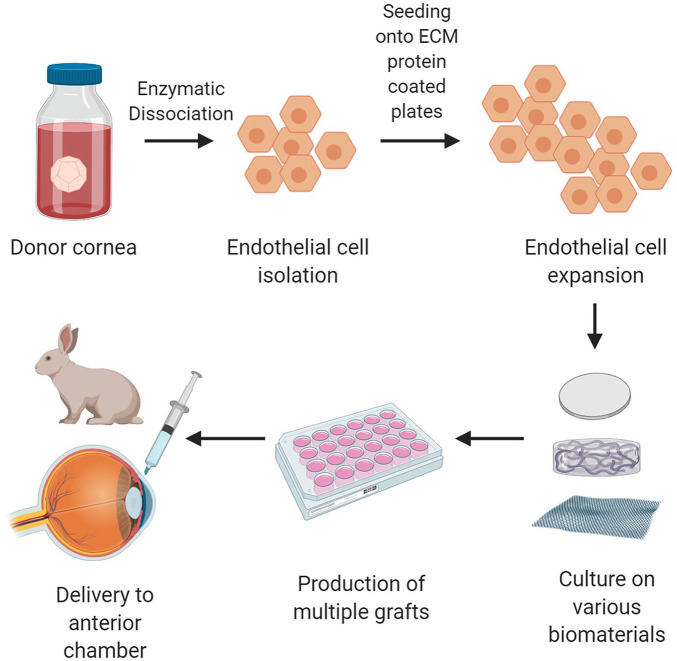
Tissue engineering of the cornea. Cadaveric donor corneas suitable for research are acquired. The hCECs are isolated as per optimized protocols and allowed to expand in number in culture. Once there are sufficient number of cells, they are transferred onto a scaffold which can be biologically derived, synthetic or semi-synthetic. Multiple grafts can then be produced as a confluent monolayer of cells on the biomaterials. These grafts can then be introduced into the anterior chamber of an animal for research purposes with the eventual goal of transplanting into humans. Figure created with BioRender.com.

An alternative approach to injected therapy is the use of cells combined with a biomaterial to create tissue engineered grafts for transplantation. This involves isolating and expanding the cells in the same way as for cell injection but then seeding them onto a scaffold to allow them to form a confluent monolayer before transplantation (Figure 4). There have been challenges associated with this approach, most notably being able to provide the specialized environment required for favorable expansion followed by maintenance of an endothelial phenotype as well as production of a robust endothelial tissue which can be handled without difficulty for transplantation.^2^ Steps have been made to combat this, including the use of different types of biomaterials, with the aim of mimicking some of the features of DM as a base or scaffold to cultivate the hCEC monolayer. These approaches have been trialed in in vivo animal models but, so far, not in humans. Here, we compare different types of biomaterials and assess their potential advantages and drawbacks, summarized in table 1.

### Biologically derived materials

#### Amniotic membrane

**Figure 5.**
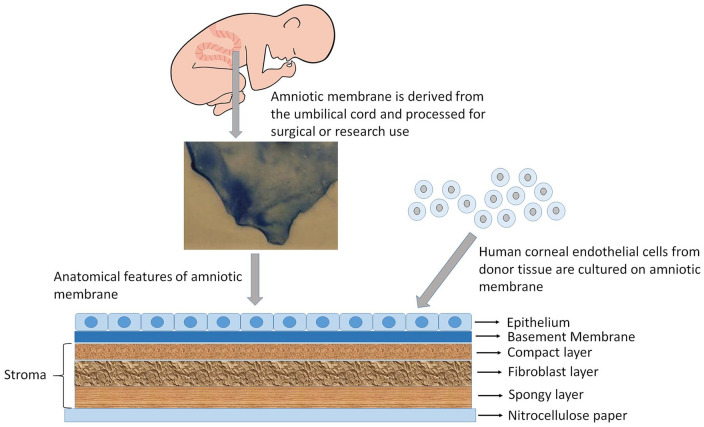
Biologically derived material. Amniotic membrane is excised from the umbilical cord, processed and preserved on nitrocellulose paper. Amniotic membrane is used for culturing corneal endothelial cells.

Amniotic membrane (AM), a natural, inert, non-cytotoxic biological material,^41^ has a major advantage as it reduces the chances of potential graft rejection due to its proven biocompatibility in ocular applications (Figure 5). Studies in cats^42^ and rabbits^43^ have also shown positive results in the maintenance of a corneal endothelial layer of appropriate thickness, which is important in allowing cells to refract incoming light. Despite its advantages, the AM has a number of limitations; including availability from a donor bank. Structurally the transparency of the CE is vital for its function,^44^ therefore the semi-opaque nature of the AM is a concern. Only one study has reported the transplantation of cultivated cat CECs using AM, where the AM becomes transparent after 6 months.^42^ The long-term graft survival suggests that its rate of biodegradability is of particular concern.^45^

Ishino et al.,^43^ investigated the morphology and structure of hCECs transplanted on denuded AM using vital staining and scanning and transmission electron microscopy. They found that the ultrastructure and density of these cells was very similar to that of normal CECs *ex vivo*, thus suggesting that AM is a suitable substrate for maintaining the endothelial phenotype in vitro. The same group transplanted hCECs on denuded AM into an in vivo rabbit model and showed that at day 7 after surgery, the graft presented flat polygonal endothelial cells with uniform size and presence of tight junctions. The corneas retained their thickness and transparency compared with the denuded AM controls for 1 week. Because rabbit CECs have been known to proliferate in vivo, the extent of survival of hCECs transplanted in vivo using DiI labeling was also studied. The results of the DiI labeling showed that the hCECs remained on the denuded AM transplanted onto the corneal button for at least 4 weeks.^43^ Even though AM appears to function well as a scaffold for hCECs, it is associated with a risk of contamination and transmission of infectious diseases. Furthermore, there is biological variability between donor tissues, sub-optimal transparency, and unpredictable degradation rates, all of which has limited its use in the context of corneal endothelial tissue engineering.^46^

#### Silk fibroin

Silk fibroin (SF), a natural fibrin derived from silk has low immunogenicity, good transparency, is non-toxic and displays a controlled degradation rate.^47^ However, it has low elasticity and so can easily present surface cracks during the production or handling processes.^48^ A 5 µm thick transparent membrane of SF was used to culture hCECs.^49^ This material can support growth of CECs, thus suggesting that it can be used as a scaffold for tissue engineering and corneal endothelial reconstruction. A silk fibroin-based artificial endothelial graft was transplanted as a DMEK into a rabbit eye.^50^ After surgery, the graft restored the corneal transparency and reduced the thickness of the cornea at 6 weeks follow-up. The rabbit CECs exhibited monolayer morphology and showed characteristic markers ZO-1 and Na+/K+-ATPase. Other studies have investigated SF modified with glycerol,^48^ β -carotene,^51^ or collagen type I,^52^ as a potential substrate for CEC regeneration. Previous studies have reported the decreased biocompatibility and increased hypersensitivity of silk-based materials due to presence of sericin.^52^ To overcome this issue, Song et al.^48^ created a silk fibroin film modified with glycerol (G/SF), used as plasticizer, and showed that G/SF films have better physical and mechanical properties, and thinner with respect to the SF films. In vitro tests showed promising results compared with the SF film: G/SF can induce an increase in CECs initial adhesion and proliferation rate when compared with the SF film.^48^ Kim et al.^51^ enhanced SF film with β-carotene (β-C). β-C, a vitamin A precursor, has been reported to enhance cell proliferation and differentiation. They showed that a proper amount of β-C incorporated with SF based film scaffolds for rabbit CECs can improve cell viability compared to SF film, moreover β-C/SF scaffolds showed more tight cell junctions and uniform cell size, and showed higher mRNA expression of Na^+^/K^+^-ATPase, aquaporin-1, chloride channel protein 3, sodium/biocarbonate co-transporter 1 and voltage-dependent anion channel 2.^51^ In vitro studies using rabbit CECs (rCECs) were conducted testing silk films surface-coated with collagen type I (Col-I/SF) to increase cell attachment. Morphological and structural characteristics were studied that showed small-sized pores and dense surface regularity values compared to the SF film, with good transparency and hydrophilicity reported. In vitro biological studies showed an increase in attachment and proliferation of cultured rCECs on the Col-I/SF films. No significant changes in the expression of ZO-1 and Na^+^/K^+^-ATPase were described.^52^ Kim et al.,^53^ used transparent ultrathin film scaffolds fabricated from aloe vera (AV) gel blended with silk fibroin. It was observed that incorporation of a small amount of AV gel increased cell viability and maintained cell function. These scaffolds were transplanted in rabbit eyes and they attached to the stroma without significant inflammatory reactions. Although the SF films show good compatibility with CECs the major drawback of SF is that the films that have been trialed in this area lack the mechanical strength required for surgical use and can be very fragile upon handling.

#### Human anterior lens capsule

**Figure 6.**
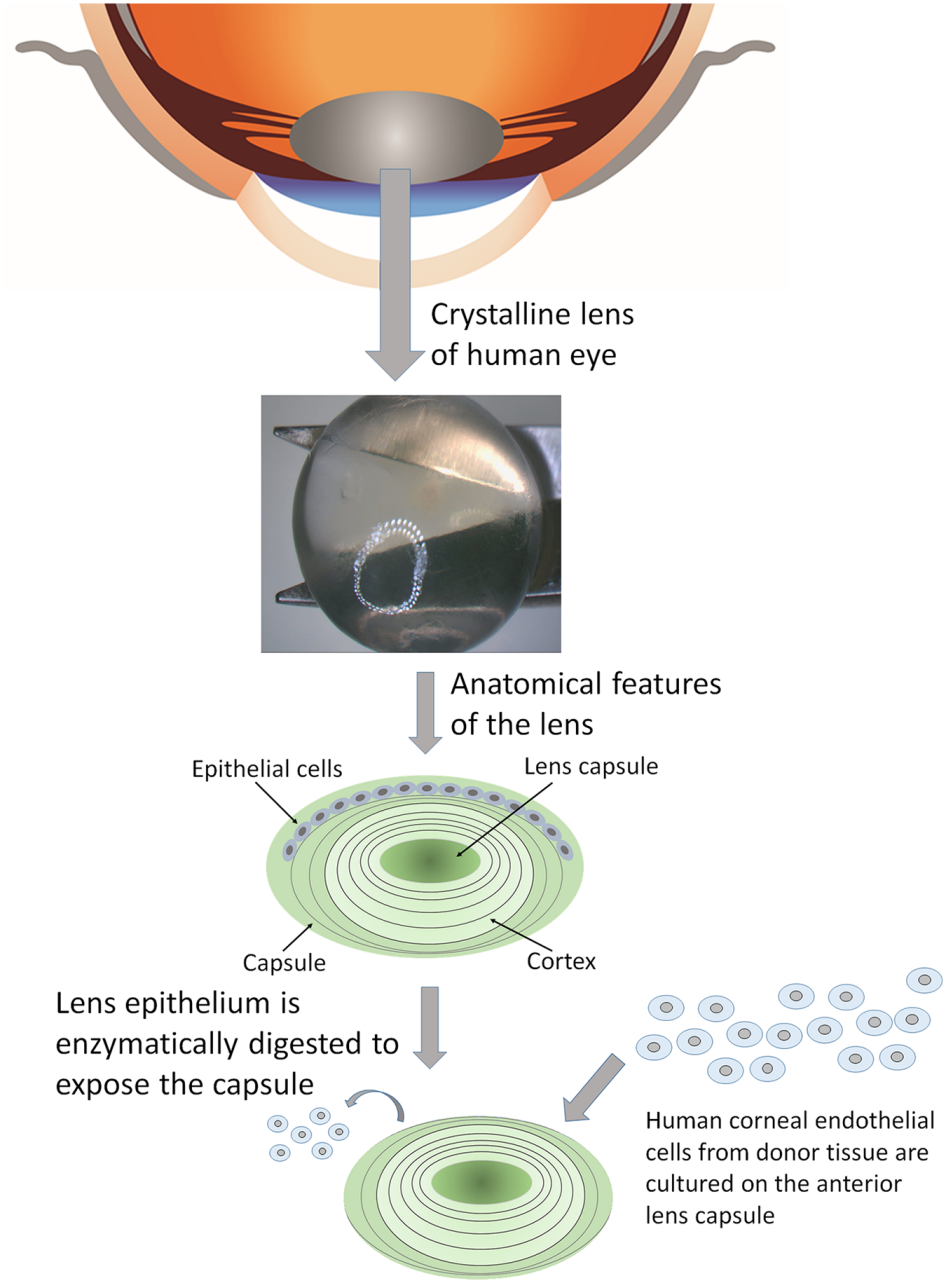
Human crystalline lens is obtained from cadaveric eye donors. The lens epithelium is enzymatically isolated and the remaining capsule is used for culturing corneal endothelial cells in vitro.

Human lens (Figure 6) is found in the anterior chamber of the eye, which refracts the surrounding light directed by the cornea towards the retina. Opacification of the lens caused due to aggregated protein deposition as a result of age can result into cataracts. The lens is clear and is covered by a smooth, transparent basement membrane called lens capsule, which is elastic and composed of type IV collagen and sulfated glycosaminoglycans. Yoeruek et al.,^54^ evaluated the potential of human anterior lens capsule (HALC) as a scaffold for cultivating and transplanting hCECs. The HALCs were obtained during cataract surgery, enzyme-digested to remove the lens epithelium and finally plated with the epithelial side up. hCECs were grown to confluence and formed a continuous viable monolayer. Immunohistochemistry showed tight junctions (ZO-1) and pump functions Na^+^/K^+^-ATPase along with expression of connexin-43 and cytokeratin 3 (AE5). HALCs were thus determined to have the capability of maintaining intact barrier and ionic pump functions in vitro. A more recent study looked at HALCs as carriers for cultivated porcine CECs.^55^ The cells formed a monolayer of hexagonal, tightly packed cells that expressed ZO-1 and Na^+^/K^+^-ATPase. In this study they also analyzed the handling ability of the grafts using a clinically applicable method. They noted that the constructs behaved in a similar way to a DMEK during implantation and unfolding in an artificial anterior chamber model, with good adhesion to a bare stroma. These physical tests are required as cell compatibility is not the only important parameter to consider. The main issue with the use of HALC as a scaffold for tissue engineering is that, being a biological material, it heavily relies on the supply of cadaveric eye donors.

#### Decellularized corneas

**Figure 7.**
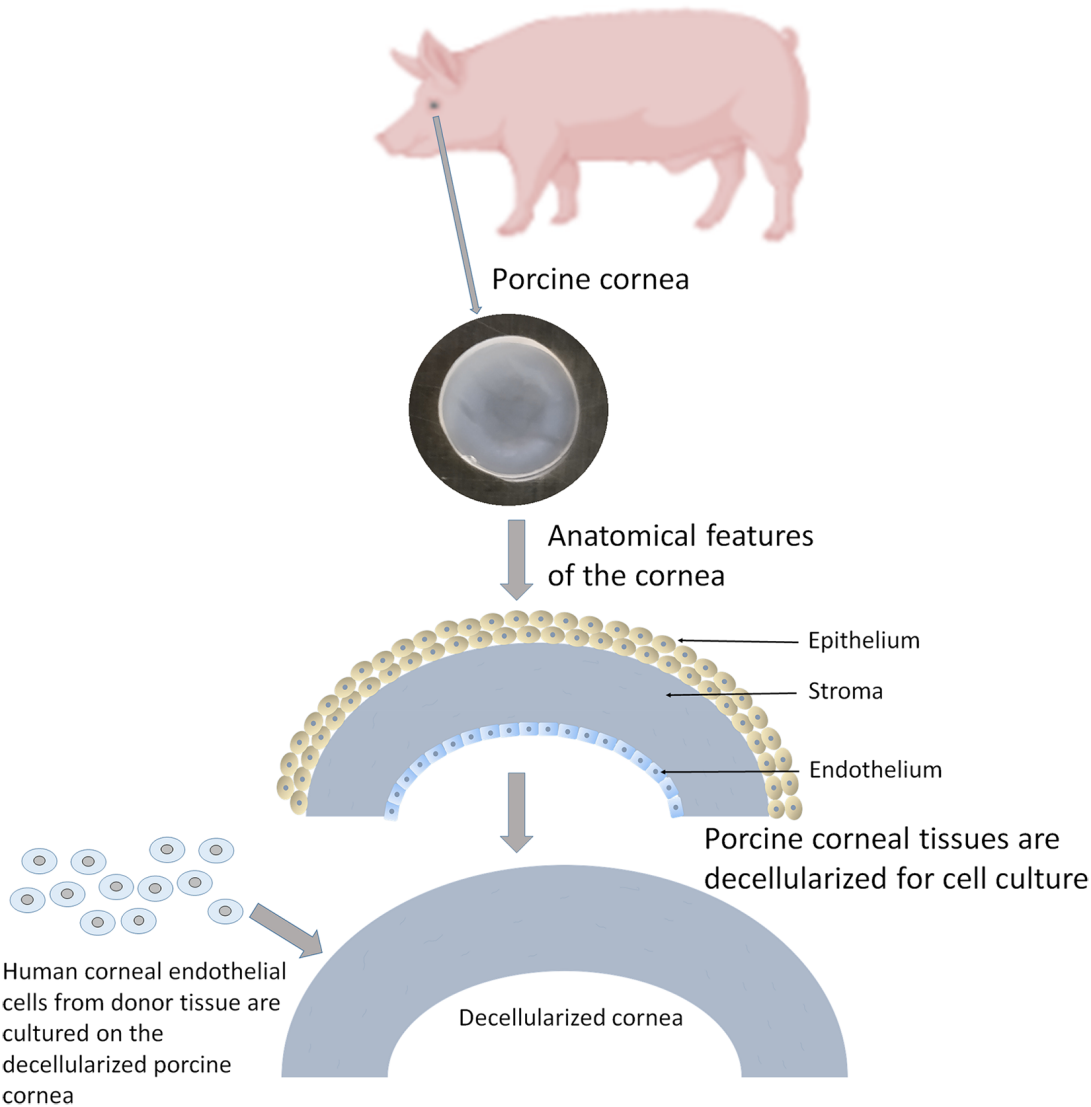
Porcine cornea is excised from the eye globe. The tissue is decellularized to preserve the stromal layer. This layer is used for culturing corneal endothelial cells in vitro.

Bayyoud et al.,^56^ evaluated the potential of decellularized bovine corneas as a carrier for cultivating and transplanting hCECs. After chemically decellularizing the bovine corneas and culturing the hCECs, it was found that bovine cells were substantially removed from the stroma and DM. A statistically significant amount of DNA reduction was observed before and after decellularization. hCECs exhibited viable, polygonal, monolayer morphology with endothelial cell density (ECD) >2300 cells/mm^2^ with expression of cytokeratin 3 (AE5), collagen type VIII, ZO-1, CX-42, NaHCo_3_, and Na+/K+-ATPase. These phenotypical properties of hCECs imply that the hCEC sheets are capable of maintaining an intact barrier and ionic pump function in vitro.^56^ Recently, acellular porcine corneas (Figure 7) were co-cultured with limbal epithelial cells and CECs derived from human embryonic stem cells.^57^ Corneal endothelial cells presented their specific markers N-cadherin, ZO-1, and Na^+^/K^+^-ATPase. After cultivation, the acellular porcine corneal scaffold was transplanted into a rabbit eye. The results showed that the tissues were well-integrated with host corneas and corneal clarity was increased without adverse effects. A recent publication has detailed the development of a method to rapidly decellularize a porcine cornea using sodium N lauroyl glutamate and supernuclease.^58^ This process allowed the removal of xenoantigen DNA within 3 h, while still retaining transparency, ultrastructure and mechanical properties of the corneas. In vivo studies confirmed there was no immune rejection and transparency was maintained. This method has shown potential to increase the appeal of the use of decellularized materials as previously the process had been criticized for being too lengthy.

#### Fish scales

In 2010, Lin et al.^59^ proposed the use of an oxygen- and glucose-permeable collagen scaffold derived from decalcified fish scales (Tilapia; *Oreochromis mossambicus*) that can be used in corneal regeneration. Until now, preliminary in vitro studies have shown cytocompatibility of corneal *epithelial* cells on these heterogeneously patterned, biological scaffolds.^60^ Its architectural features have been suggested as an important characteristic for corneal epithelial cell migration and growth. Moreover, its transparency and availability, i.e. roughly 200 scales from one fish, makes it an attractive biocompatible material for generation of corneal epithelial cell grafts. Additional in vivo studies performed on rats and rabbits have demonstrated its potential as a deep anterior lamellar keratoplasty (DALK) alternative or to seal perforated corneas, respectively.^61^ However, a recent report suggested that the fish scale derived scaffold in its current form may not be ideal for the development of tissue engineered corneal endothelial constructs.^62^ Although hCECs adhered to the surface of the scales, regional differences in cellular proliferation were observed. The morphology of the cells was also inconsistent with some areas appearing nicely cobblestone but others showing more fibroblastic-like phenotypes. Further modification of the substrate, such as surface polishing to remove the irregular topography, overall thinning of the scaffold and coating with ECM proteins such as fibronectin, could drastically improve its geometric and physical characteristics whilst also enhancing cell-matrix interactions. Post-modification fish scale scaffolds do show some promise due to their inherent transparency once de-calcified and good mechanical properties allowing easy folding like DSAEK grafts.

#### Plastic compressed collagen

**Figure 8.**
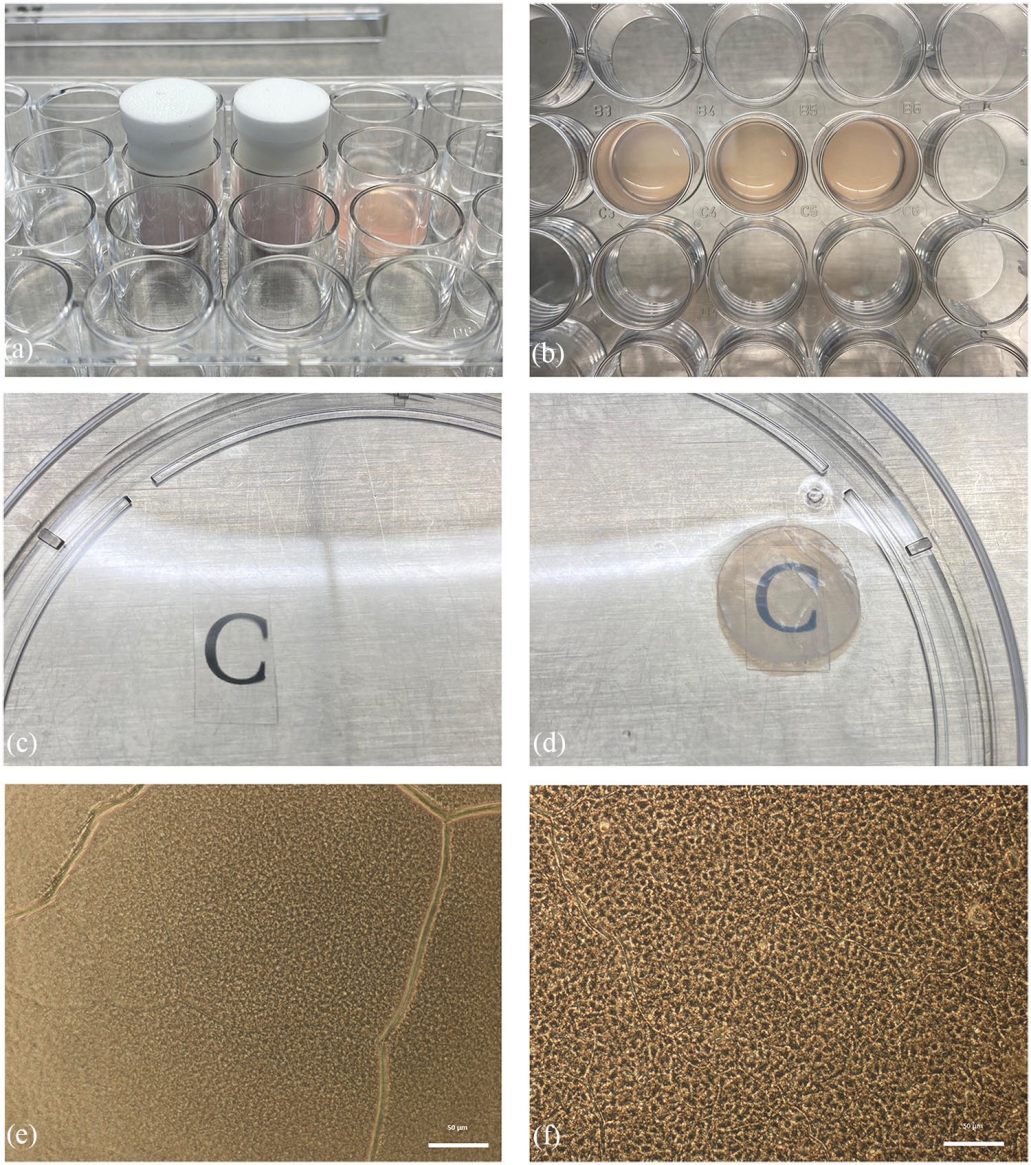
Preparation of Real Architecture for 3D Tissues (RAFT) scaffolds: (a) collagen mixture is pressurized using a plunger to form (b) RAFT scaffolds. The transparency is checked using (c) a transparent slide as a control and compared with (d) the RAFT scaffold. RAFT, as observed under light microscope (e) without cells, and (f) with cultured corneal endothelial cells in vitro.

Levis et al.^63^ described the use of a Real Architecture for 3D Tissues (RAFT) biomaterial that is based on the process of plastic compression of collagen type I. RAFT is a simple and rapid method of production, which yields multiple reproducible constructs with limited variability between batches. The process to manufacture RAFT requires collagen and a plunger (Figure 8(a)) to pressure-build the scaffold (Figure 8(b)). Moreover, it was noted that RAFT is a superior biomaterial in terms of its transparency (Figure 8(c) and (d)), mechanical strength as it is sufficient to withstand the manipulation that would be required for transplantation without the need for any chemical crosslinking that may have deleterious effects on the behavior of cells on the surface or after transplantation. RAFT is shown to be a highly effective, novel carrier for hCECs (Figure 8(e)). Both hCEC lines and primary hCECs retained their morphologic and molecular characteristics for up to 14 days when they were cultured on these scaffolds (Figure 8(f)).^64^ This was demonstrated by the successful loading and delivery of a RAFT with hCECs to an *ex vivo* porcine eye using a clinical insertion device.

#### Gelatin

Gelatin is a potentially useful biomaterial in tissue engineering applications as it is chemically similar to ECM proteins, has low antigenicity, cost effectiveness, abundance, and accessible functional groups that can allow chemical modifications.^65^ Kimoto et al.^66^ evaluated novel bioengineered CEC sheets based on a gelatin hydrogel that fit to the curvature of the posterior corneal surface. Monkey CECs were seeded on the hydrogels and the cells were examined by immunohistochemistry after transplanting these scaffolds into a monkey model of bullous keratopathy. These sheets showed permeability to water and protein similar to that of atelocollagen and vitrigel sheets. Monkey cells expressed tight junction proteins and ion pumps were normal, N-cadherin was also expressed. Transparency and thickness was maintained. It was noted that these spherically curved gelatin hydrogel sheets achieved close adhesion to the posterior corneal surface without wrinkling and may be useful clinically. It is worth noting, however, that hCECs were not used in that study so their compatibility with the material needs to be tested.

Niu et al.^67^ prepared a series of transparent gelatin fabricated scaffolds modified with heparin. It was observed that heparin-modified scaffolds displayed a greater capacity to absorb basic fibroblastic growth factor (bFGF) and showed better release kinetics for up to 20 days. It was also noted that the release of bFGF from the scaffolds improved the survival of hCECs and further reduced cellular loss. An in vivo animal study showed that these scaffolds were flexible enough to be folded and implanted in rabbits’ eyes through a small incision in the cornea. The scaffolds adhered to the inner surface of the corneal stroma and gradually integrated with the surrounding tissue. Thus, the results indicate that gelatin based corneal scaffolds modified to absorb and release growth factors and seeded with hCECs could be useful for transplantation purposes.

#### Chitosan blends

Chitosan is a biomimetic polysaccharide derived from chitin, a component of the exoskeleton of crustaceans. It has high biocompatibility and biodegradability, but low strength, therefore, to enhance its suitability for this application it was combined with two other naturally occurring materials. A polymer that consisted of hydroxyethyl chitosan, gelatin, and chondroitin sulfate was created.^68^ In terms of cultivation of hCECs on the substrate, results were encouraging with a significant increase in cell proliferation seen by day 4, and both the membrane permeability and cell organization were comparable to the native human cornea. It was then inserted into the anterior chamber of the rabbit models, where after 3 weeks, the cornea retained its clarity and structure. Unfortunately, inflammation was noticed initially at the corneal-iris junction but no information was provided about ECD at 3 weeks, which is important when considering that inflammation can lead to endothelial cell loss (ECL). Whilst these results look promising, this formulation is still in the experimental stage with more work required to ascertain its suitability for human use.

### Synthetic materials

#### Poly-ε-lysine peptide hydrogel

**Figure 9.**
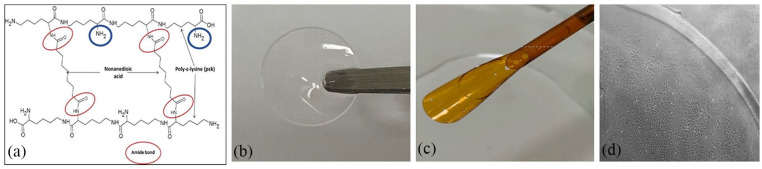
Poly-ε-lysine peptide hydrogels: (a) the poly-ε-lysine backbone structure is cross-linked with diacid forming amide bonds (red circles) leaving free amine sites which can be functionalized (blue circles), (b) the hydrogels are highly transparent and (c) have mechanical strength to allow loading into an endothelial graft delivery device, and (d) porcine CECs attach and expand and form a monolayer on the surface of the gel.

Kennedy et al.^69^ proposed the use of a synthetic peptide hydrogel using poly-ε-lysine (pεK), cross-linked with octanedioic-acid as a potential substrate for CEC expansion, using hCEC lines and primary porcine endothelial cells. A human corneal endothelial cell line (HCEC-12) attached and grew on pεK hydrogels as confluent monolayers after 7 days, while porcine CECs (pCECs) required functionalization of the pεK hydrogel with a synthetic cell binding peptide to adhere. This resulted in enhanced pCEC adhesion and growth, with expression of ZO-1 and Na^+^/K^+^-ATPase at 5 weeks, suggesting a functional corneal endothelial layer.^69^ The advantage of synthetic biomaterials is that their properties can be tightly controlled to produce a customized material. This particular biomaterial also has a number of free amine sites allowing functionalization of the surface with synthetic peptides if required, which make it even more customizable (Figure 9(a)). The mechanical properties and transparency can also be modified depending on the formulation, percentage crosslinking, and type of crosslinker. The formulation has been optimized to provide high transparency, mechanical properties to allow loading and delivery from a clinical graft delivery device and adhesion, and expansion of primary cells on the surface (Figure 9(b)–(d)) This synthetic peptide shows great potential to be a suitable scaffold to create a tissue engineered corneal endothelial graft.

#### Poly(methyl-methacrylate), poly(lactic-co-glycolic acid), and polycaprolactone

In the last few years there has been a mounting interest in the fabrication of tissue-engineered scaffolds by a process referred to as electro-spinning. Kruse et al.^70^ tested the cultivation of human CECs on electro-spun scaffolds, obtained from three different synthetic polymers: poly(methyl-methacrylate) (PMMA), poly(lactic-co-glycolic acid) (PLGA), and polycaprolactone (PCL). Cultivation was examined after 3–7 days of incubation: PMMA showed significant cytotoxicity, while PLGA and PCL electro-spun scaffolds resulted in equal biocompatibility (tested by luminescence assay) but only PLGA maintained hCEC morphology. The three polymer solutions differed significantly in their viscosity. The lowest viscosity amongst the three polymer solutions was found in PMMA, which produced the largest diameter fibers and higher thickness confirmed using optical light microscope measurements. The PLGA fibers, being the thinnest, were densely packed, so that the hCECs could grow on the surface and maintain their morphology.^70^

As well as controlling fiber morphology, electrospinning can be used to fabricate specific gross morphology and control the fiber orientation of tissue engineered scaffolds. A study by Kim et al.^71^ using PCL and collagen type I found the mechanical properties of the radially aligned section of the scaffolds were supported by the randomly orientated sections making the substrate adequate for corneal application. Transparency of the wetted radially aligned scaffolds was comparable to native rabbit cornea between the 400 and 800 nm wavelength range. Cell culture results showed primary rabbit corneal cells (isolated epithelial, endothelial, keratocytes, and limbal stem cells) exhibited significantly higher proliferation rates with significantly higher cell migration rates across the surface of radially aligned fibers compared to randomly orientated fibers. Additionally, they found CECs cultured on radially aligned fibers showed significantly greater expression of ZO-1 and Na^+^/K^+^-ATPase compared to when cultured on randomly orientated fibers.^71^

### Semi-synthetic materials

#### Gelatin methacrylate

Both, natural and synthetic polymers have their drawbacks, therefore, combinations of biological derived and synthetic materials have attracted attention in the field of tissue engineering. Studies have shown gelatin methacrylate (GelMA) could be useful as a biomaterial with applications in corneal tissue engineering, but evidence of sufficient strength for corneal tissue transplant is not clear as clinically relevant transplant simulations have not been carried out with this material.^72^ Rizwan et al.^19^ produced a modification of GelMA to employ it as scaffold for a tissue engineered hCEC monolayer. They developed a sequential hybrid crosslinking process (physical followed by UV crosslinking) to create an improved material, named GelMA+. GelMA+ showed an 8-fold increase in mechanical strength as compared to regular GelMA and slower degradation kinetics in vitro and in vivo. They achieved hydrogel patterning of topographical cues in the range of 1 µm or lower, that improved cell growth and viability. hCEC monolayers grown on GelMA+ scaffolds showed ZO-1 expression, higher cell density and cell size homogeneity compared to GelMA, which are indications of functionally superior monolayers.

#### Chitosan plus polycaprolactone or PEG

Chitosan is a biomimetic polysaccharide and PCL is a biodegradable polyester and both are biomaterials approved by the Food and Drug Administration (FDA). Wang et al.^73^ hybridized chitosan and polycaprolactone to create blended membranes. Chitosan has low strength, whereas PCL has good mechanical properties, and the ability to promote cell adhesion and ECM production. The blended membrane was reported to be beneficial in allowing the growth of CECs, and in maintaining their phenotype in vitro. After 14 days of incubation on 25% PCL and 75% chitosan, CECs showed expression of the tight-junction ZO-1, the Na^+^/K^+^-ATPase, and the gap junction protein connexin-43, which were examined to confirm the functional features of the cells. In a more recent study, the same group showed that hCECs cultured on the blended membranes were able to lay down their own ECM matrix containing collagen IV, which is present in DM.^74^

Chitosan has also been combined with poly(ethylene glycol) (PEG) to form ultrathin hydrogel films.^75^ The mechanical properties such as tensile strain and ultimate stress were identical to or greater than those of human corneal tissue due to the fine tuning of the PEG content. Transparency of the films was excellent and they were able to support the attachment and proliferation of sheep CECs. Importantly, they were also subjected to physical manipulation in *ex vivo* surgical trials on ovine eyes, in which they performed well.

## Emerging technologies

### Electrospun/electrosprayed bioactive materials

**Figure 10.**
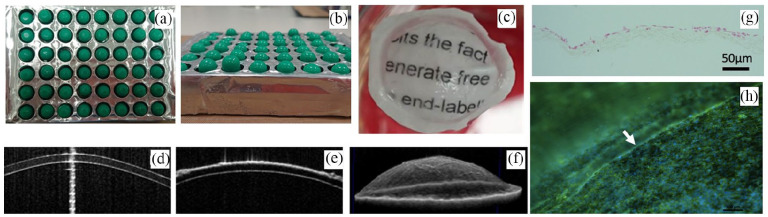
CECs on electrospun PET membranes: (a) and (b) patterned plate used to collect eletrospun fibers, (c) wet membrane showing good transparency and conformity to the surface of a hemispherical contact lens, (d) optical coherence tomography images of (d) a contact lens alone, (e) a contact lens with the membrane on the anterior surface and (f) a side view showing the conformity of the membrane to the surface of the contact lens, (g) hematoxylin and eosin stained section of the membrane after 4 weeks of cell culture with HCEC-12 cells, and (h) DAPI and phalloidin staining of the HCEC-12 cells on the surface of the membrane showing a preference for the central region. White arrow indicates the start of the opaque frame of the membrane.

Bioactive electrospun materials loaded with drugs to prevent vascularization of the cornea have been investigated.^76^ Silk nanofibers loaded with epigallocatechin gallate (EGCG) exhibited a dose-dependent inhibition of human umbilical vein endothelial cell proliferation. Similarly, nanoparticles fabricated by electrospraying to encapsulate biologically active compounds decorated on electrospun membranes are also being investigated for the corneal endothelium. The idea being that the substrate will act two-fold, to deliver cells to the required area while delivering drugs/growth factors to the eye long-term to maintain transplanted cell health.^77^ Preliminary studies using polyethylene terephthalate (PET), an FDA approved non-degradable polymer, electrospun from a solution dissolved in 1,1,1-3,3,3-hexafluoroisopropanol, are described here. Fibers were collected on a grounded, static collection plate modified with insulated sections (Figure 10(a) and (b)). The insulating sections were made of cast molded polydimethylsiloxane (PDMS) in a hemispherical shape. The collected fibers formed a transparent membrane on the insulated sections, whereas the fibers collected on the conducting areas formed an opaque frame. Wetting improved the transparency of the membranes (Figure 10(c)). Wetted membranes mounted on a standard contact lens imaged using optical coherence tomography highlight the close approximation of the membrane on the anterior surface of the lens (Figure 10(d)–(f)). Culturing a human corneal endothelial cell line (HCEC-12) on the surface of the membranes for 1 month indicated the cytocompatibility of the membranes, without any requirement for further surface functionalization. Cells did not invade the bulk structure of the membrane, shown in histological sections (Figure 10(g)) and showed a distinct preference for the transparent area of the membranes in comparison to the opaque frames (Figure 10(h)).

### Additive manufacturing

**Figure 11.**
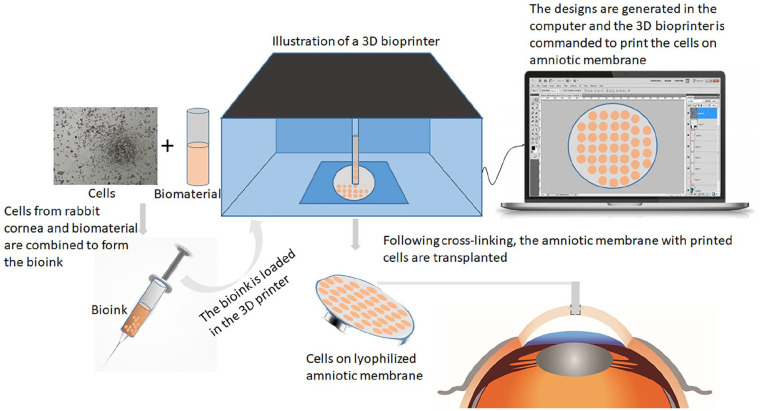
Illustration of 3D bioprinting. The cultured cells are expanded and mixed with the biomaterial to form the bioink. This bioink is then loaded into the printer. Computer program helps to design the final product, which is then printed in the 3D bioprinter on the desired scaffold. The final model is thus generated after cross-linking and transplanted.

Since its invention, additive manufacturing, or three-dimensional (3D) printing is being exploited in innovative new ways in applications in engineering, manufacturing, education, art, and medicine. It has caught the attention of tissue engineers due to the possibilities of 3D bioprinting which allows the incorporation of living cells into the printing ink. The process is complex with choice of printing type, materials, cell types, growth, and differentiation factors as well as technical issues with maintaining cell survival and construction formation.^78^ Focus on corneal bioprinting has predominantly been on creation of corneal stromal tissues either for transplantation or for use as an in vitro model.^79,80^ A recent study explored the use of 3D-bioprinting to rapidly deposit hCECs onto amniotic membrane to produce a graft for transplantation into a rabbit model of endothelial damage (Figure 11).^81^ The cells were deposited onto lyophilized amniotic membrane using extrusion in a gelatin based bioink supplemented with 0.02 % arginylglycylaspartic acid (Arg-Gly-Asp; RGD), a cell adhesion peptide. The structure was designed as 8 mm× 8 mm with a thickness of 0.7 mm and crosslinked with ultraviolet light for 15 s. The hCECs were shown to survive the printing process and attach to the amniotic membrane. The appearance of the hCECs was atypical with no evidence of a confluent, cobblestone monolayer after 10 days in culture, however, in the rabbit model the graft group did show improvement of corneal clarity after 2 weeks and near complete clarity by 4 weeks. In this study, another biomaterial, amniotic membrane, was required as a base for the printing. When the technology is developed so that the bio-ink produced scaffold alone can be transplanted then this could increase the advantages of this method of graft creation for CECs. The true potential of this application in the field of corneal endothelial tissue engineering is still unknown as there are still many parameters to be optimized.

## Conclusion

A huge amount of progress has been made towards development of a tissue engineered corneal endothelial graft. Bioengineering a scaffold material offers some advantages over biologically derived materials as variability can be controlled, materials can be tuned based on their desirable properties to further increase the reproducibility and enable mass production when necessary with batch-batch consistency. A number of different cell biomaterials have been studied for the purpose of endothelial layer construction, but success is limited by the specific requirements of a scaffold material in this context. These include; cytocompatibility, reproducibility, ease of production/supply, transparency, ability to be handled easily by surgeons i.e. flexibility ideally with tunable properties such as thickness. Many of the publications involving scaffold based engineered grafts have only progressed as far as in vitro or in vivo studies and first-in-human studies are very limited. A big challenge is to acquire suitable funding to progress the potential novel tissue engineered grafts along the translational pathway to clinical trial. Even if a particular material is successful in vitro or in vivo, adjusting the manufacturing protocol to comply with good manufacturing practice (GMP) standards and to allow scale up can be challenging. Throughout the development process researchers should be in contact with the relevant regulatory bodies to ensure the correct safety and efficacy studies have been carried out to allow the product to progress towards the treatment of patients.
